# Frequency of Effector Memory Cells Expressing Integrin α_4_β_7_ Is Associated With TGF-β1 Levels in Therapy Naïve HIV Infected Women With Low CD4^+^ T Cell Count

**DOI:** 10.3389/fimmu.2021.651122

**Published:** 2021-03-22

**Authors:** Nandini J. Kasarpalkar, Shilpa Bhowmick, Vainav Patel, Lalita Savardekar, Sachee Agrawal, Jayanthi Shastri, Vikrant M. Bhor

**Affiliations:** ^1^Department of Molecular Immunology and Microbiology, Indian Council of Medical Research-National Institute for Research in Reproductive Health, Mumbai, India; ^2^Department of Biochemistry and Virology, Indian Council of Medical Research-National Institute for Research in Reproductive Health, Mumbai, India; ^3^Woman's Health Clinic and Bone Health Clinic, Indian Council of Medical Research-National Institute for Research in Reproductive Health, Mumbai, India; ^4^Department of Microbiology, Topiwala National Medical College and Bai Yamunabai Laxman Nair Hospital, Mumbai, India

**Keywords:** integrin α_4_β_7_, HIV, soluble MAdCAM-1, TGF-β1, effector memory cells

## Abstract

Integrin α_4_β_7_ expressing CD4^+^ T cells are preferred targets for HIV infection and are thought to be predictors of disease progression. Concurrent analysis of integrin α_4_β_7_ expressing innate and adaptive immune cells was carried out in antiretroviral (ART) therapy naïve HIV infected women in order to determine its contribution to HIV induced immune dysfunction. Our results demonstrate a HIV infection associated decrease in the frequency of integrin α_4_β_7_ expressing endocervical T cells along with an increase in the frequency of integrin α_4_β_7_ expressing peripheral monocytes and central memory CD4^+^ T cells, which are considered to be viral reservoirs. We report for the first time an increase in levels of soluble MAdCAM-1 (sMAdCAM-1) in HIV infected individuals as well as an increased frequency and count of integrin $\beta_{7}^{\text{Hi}}$ CD8^+^ memory T cells. Correlation analysis indicates that the frequency of effector memory CD8^+^ T cells expressing integrin α_4_β_7_ is associated with levels of both sMAdCAM-1 and TGF-β1. The results of this study also suggest HIV induced alterations in T cell homeostasis to be on account of disparate actions of sMAdCAM-1 and TGF-β1 on integrin α_4_β_7_ expressing T cells. The immune correlates identified in this study warrant further investigation to determine their utility in monitoring disease progression.

## Introduction

The assault of Human Immunodeficiency virus (HIV) on the immune system is primarily reflected in the decline in absolute count of CD4^+^ T cells as well as the ratio of CD4/CD8 T cells (1). The latter is a result of not just CD4^+^ T cell depletion but also a concurrent expansion of CD8^+^ T cells (2). Homeostasis of T cell subsets is also altered during HIV infection (3). In addition to T cells, immune dysfunction i.e., altered signaling leading to impaired proliferation and anti-viral responses, is also seen in B cells (4), monocytes (5, 6), dendritic cells (7) and Natural killer (NK) cells (8, 9) following HIV infection. This dysfunction is attributed to both the direct interaction of immune cells with the viral components as well as altered levels of pro- and anti-inflammatory cytokines. Besides these factors, HIV acquisition as well as disease progression also depends on the level of expression of HIV co-receptors such as CCR5 (10–12) and attachment receptors such as integrin α_4_β_7_ (13, 14) on CD4^+^T cells.

Interaction of HIV envelope protein gp120 with integrin α_4_β_7_ on CD4^+^ T cells results in downstream signaling events which facilitate infection (15, 16) making them preferred targets for HIV infection (17, 18). Gut homing property of integrin α_4_β_7_ also contributes toward establishing HIV infection in the gut associated lymphoid tissue (GALT) (19), which is severely and irrevocably damaged upon HIV infection (20–23). Additionally, interaction of integrin α_4_β_7_ with the form of its natural ligand, Mucosal Addressin cell adhesion molecule-1 (MAdCAM-1) results in T cell activation and proliferation, promoting HIV replication (24, 25). Recent evidence suggests the formation of latent HIV reservoirs through differentiation of HIV infected integrin α_4_β_7_ expressing effector memory T cells into central memory cells *in vitro* under the influence of transforming growth factor β (TGF-β) (26). Integrin α_4_β_7_-HIV gp120 interaction has also been targeted using monoclonal antibodies for prophylactic as well as therapeutic applications in both animal models (27–30) and human studies (31).

The frequency of integrin α_4_β_7_ expressing cells varies among T cells subsets (32) as well as other immune cells in healthy and diseased states (31, 33, 34). Moreover, interaction of HIV gp120 with integrin α_4_β_7_ on different immune cells also hampers their function (35–37). Expression of integrin α_4_β_7_ on different immune subsets has been studied in non-human primates (38) as well as humans (39) but few studies have focused on factors that contribute toward changes in the frequency and counts of these cells during HIV infection.

In the present study, we examined the distribution of integrin α_4_β_7_ expressing immune cells in antiretroviral therapy naïve HIV infected women. We also examined their association with levels of soluble MAdCAM-1 (sMAdCAM-1) and TGF-β1. The results suggest that sMAdCAM-1 and TGF-β1 mediated immunomodulation contributes to dynamic changes in frequencies and counts of integrin α_4_β_7_ expressing immune cells and these in turn may influence disease progression in therapy naïve HIV infected women.

## Results

Women in the age group of 18–45 years and having a regular menstrual cycle were recruited from the integrated counseling and testing center (ICTC) of a tertiary care hospital as well as the women's health clinic of ICMR-NIRRH, Mumbai, India. Clinical characteristics of study participants (*n* = 48) are given in Table 1. The participants were segregated into two groups depending on their HIV status with HIV infected group comprising of 27 women and HIV-uninfected group comprising of 21 women. All HIV infected women were recently diagnosed and their samples were collected prior to initiation of antiretroviral therapy. Median age and day of sample collection post-first day of last menstrual period (LMP) were comparable between both the groups. HIV-uninfected study participants were apparently healthy women who were found to be negative for HIV, HSV-2 and bacterial vaginosis as well as co-infections such as hepatitis B, hepatitis C and syphilis. All the HIV-1 infected participants were also found to be negative for hepatitis B, hepatitis C and syphilis infection. Their status with respect to HSV-2 and bacterial vaginosis is summarized in Table 1.

**Table 1:** Characteristics of study population.

**Demographics**	**HIV^**+**^**	**HIV^**−**^**
N	27	21
Age (years)^a^	35 (29–49)	39 (34–41)
LMP^*^ (days)^a^	13 (8–23)	19 (16.5–23.5)
HSV serum IgG positive	16	0
Bacterial vaginosis (BV)	5	0
Nugent score > 7		
CD4 count^a^	423 (204–576)	Not done

a *Data is expressed as median (interquartile range)*.

* *Day of sample collection from first day of last menstrual period*.

### Frequency of Integrin α_4_β_7_ Expressing Cells Is Higher Among Adaptive Immune Cells in HIV Infected Women

**Figure 1:**
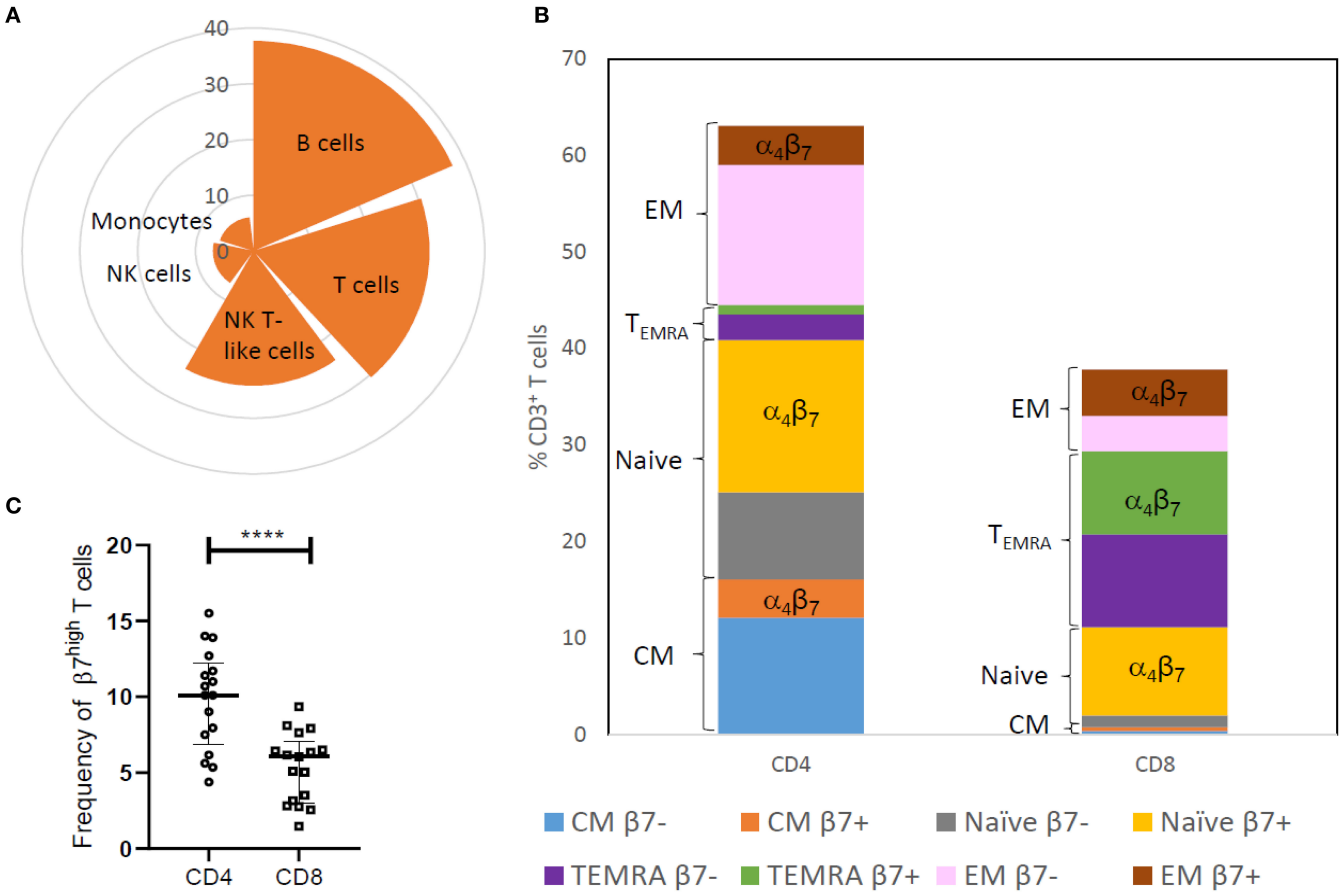
Distribution of integrin α_4_β_7_ on innate and adaptive immune cells in HIV-uninfected (HIV^−^) women. **(A)** Nightingale rose plot shows the median frequency of integrin α_4_β_7_ expressing cells among B cells, T cells, natural killer (NK) cells, monocytes and NK T-like (NKT) cells in peripheral blood (*N* = 21). **(B)** The stacked bar represents distribution of CD3^+^T cell subsets like naïve cells, effector memory cells (EM), central memory cells (CM) and terminally differentiated memory cells expressing RA (TEMRA) and median frequency of cells expressing β_7_ within these subsets (*N* = 17). **(C)** Frequency of memory CD45RA^−^CD4^+^ T cells and CD45RA^−^CD8^+^ T cells having high expression of integrin β_7_ is depicted (*N* = 17). Median and interquartile range (error bars) are depicted. Statistical analysis was performed by Wilcoxon matched-paired signed rank test using Graphpad Prism 8. ****p < 0.00001.

The proportion of discrete immune cells expressing integrin α_4_β_7_ in peripheral blood of HIV-uninfected Indian women was assessed using the BD Accuri C6 flow cytometer. Two panels each comprising of four different fluorophore tagged antibodies including both integrin α_4_ and integrin β_7_ antibodies were designed (Supplementary Figure 1). In line with previous studies, we found frequency of cells expressing integrin α_4_β_7_ to be highest among adaptive immune cells such as B cells [Median = 37.69, interquartile range (IQR = 30.56–57.79)] and T cells [Median = 30.45, (IQR = 23.83–38.46)] compared to innate immune cells such as monocytes [Median = 6.07, (IQR = 2.80–11.55)] and natural killer (NK) cells [Median = 6.99, (IQR = 2.56–9.99)]. The frequency of cells expressing integrin α_4_β_7_ (Figure 1A) was observed to be intermediate among natural killer T -like cells [Median = 24.17, (IQR = 13.88–31.25)] which display properties of both innate cells (NK cells) and adaptive cells (T cells). As previously reported (17, 40), we also found that all peripheral immune cells expressing integrin β_7_ also expressed integrin α_4_. Hence the panels designed subsequently, used integrin β_7_ as a marker for integrin α_4_β_7_ to probe the frequency of T cell subsets expressing integrin α_4_β_7_ (Supplementary Figure 2). T cell subset analysis revealed a higher frequency of integrin β_7_ expressing cells among cytotoxic CD8^+^ T cells [Median = 59, (IQR = 52.55–68.80)], compared to the helper CD4^+^ T cells [Median = 39.9, (IQR = 29.45–48.3)] (Figure 1B). Taken together, these observations indicate that higher frequency of integrin α_4_β_7_ expressing cells was observed among subsets that are actively involved in mounting targeted immune responses against the pathogen.

Naïve T cells, which develop into memory cells upon encountering pathogens, exhibited the highest frequency of integrin β_7_ expressing cells among CD4^+^ T cells [Median = 62.4, (IQR = 53.9–79.3)] as well as CD8^+^ T cells [Median = 86.8, (IQR = 84.7–92.2)]. However, as previously reported (13), naïve cells have a moderate expression of integrin β_7_ compared to the high expression observed on some CD45RA^−^ memory T cells. Higher frequency of these CD45RA^−^ $\beta_{7}^{\text{Hi}}$ T cells was observed in CD4^+^ T cells compared to CD8 T cells (Figure 1C and Supplementary Figure 3) most likely on account of lower frequency of CD45RA^−^ memory T cells in the CD8 compartment.

### Potential HIV Reservoirs Have Higher Frequency of Integrin α_4_β_7_ Expressing Cells

**Figure 2:**
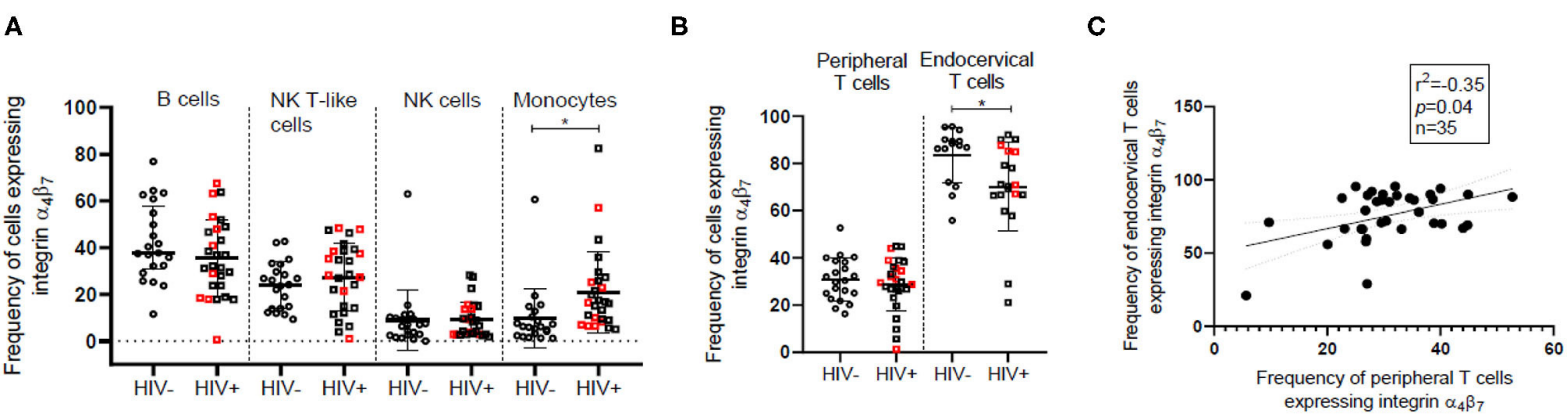
Comparison of the frequency of integrin α_4_β_7_ expressing innate and adaptive immune cells in HIV-uninfected (HIV^−^) and HIV infected (HIV^+^) women. **(A)** Median frequency and distribution of integrin α_4_β_7_ expressing B cells, natural killer (NK) cells, monocytes and NK T-like (NKT) cells in peripheral blood of HIV^−^ (*N* = 21) and HIV^+^ (*N* = 27) women. Circles represent HIV^−^ women and squares represent HIV^+^ women. **(B)** Frequency of integrin α_4_β_7_ expressing T cells in peripheral blood and endocervix of HIV^+^ (*N* = 15) and HIV^−^ (*N* = 20) women. Red squares indicate HIV^+^ women with peripheral CD4^+^ T cell count > 500. Median and interquartile range (error bars) are depicted. Statistical analysis was performed by Mann-Whitney-test using Graphpad Prism 8. *p < 0.05 **(C)** Spearman correlation between frequency of CD3^+^T cells expressing integrin α_4_β_7_ in the endocervix and peripheral blood. Significance of the correlation is represented by *p*-value and the degree of association is represented by *r* square (*r*^2^) while n represents sample size.

In view of the importance of the GALT in HIV infection, we have compared the absolute counts and frequency of the cells expressing the gut homing receptor, integrin α_4_β_7_ among different peripheral immune cell subsets between HIV infected and HIV-uninfected women. We observed increased frequency of integrin α_4_β_7_ expressing cells among innate immune cells in peripheral blood of HIV infected women with the differences being statistically significant (*p* = 0.004) in the monocyte subset {HIV infected [Median = 16.48, (IQR = 8.93–25.82)]; HIV-uninfected [Median = 6.07, (IQR = 2.80–11.55)]} (Figure 2A). However, the median frequency of integrin α_4_β_7_ expressing cells remained unperturbed among adaptive immune cells including T cells despite the loss of T cells during HIV infection. Moreover, absolute numbers of integrin α_4_β_7_ expressing B, NK, and NKT cells remained unchanged in individuals with HIV infection. The absolute counts of integrin α_4_β_7_ expressing CD4^+^ T cells were lower and those of CD8^+^ T cells were higher in HIV infected individuals despite no change in the counts of the total T cells expressing integrin α_4_β_7_ (Supplementary Figure 4). Mucosal T cells present in the endocervix were found to have a higher frequency of integrin α_4_β_7_ expressing cells compared to their peripheral counterparts in both HIV-uninfected [Blood, (*n* = 21) Median = 30.45, (IQR = 23.83–38.46); endocervix, (*n* = 15) Median = 87.5, (IQR = 72–90)] and HIV infected [Blood, (*n* = 27) Median = 29.57, (IQR = 26.05–38.29); endocervix, (*n* = 20) Median = 70.60, (IQR = 66.45–85.13)] women (Figure 2B). It is not clear if the differences observed in frequencies of T cells expressing integrin α_4_β_7_ can be attributed to overall variances in distribution of naïve and memory T cell phenotypes at the mucosal and peripheral sites (41). However, observed frequencies of T cells expressing integrin α_4_β_7_ were correlated in blood and endocervix supporting the earlier findings that reported similar correlation in $\beta_{7}^{\text{Hi}}$ cells (13) (Figure 2C). Mucosal T cells present in the endocervix are reported to be depleted concurrently with peripheral T cells following HIV infection (41) and explains the lower frequency of endocervical T cells expressing integrin α_4_β_7_ among HIV infected women [Median = 70.6, (IQR = 66.45–85.13)]; in comparison to HIV-uninfected women [Median = 87.5, (IQR = 72–90)] (Figure 2B).

Fifty-seven percentage of the HIV infected women were also positive for latent HSV-2 infection as indicated by presence of IgG antibodies against HSV-2. However, frequency of cells expressing integrin α_4_β_7_ in HIV infected women did not vary significantly in the presence and absence of HSV-2 coinfection (Supplementary Figure 5).

**Figure 3:**
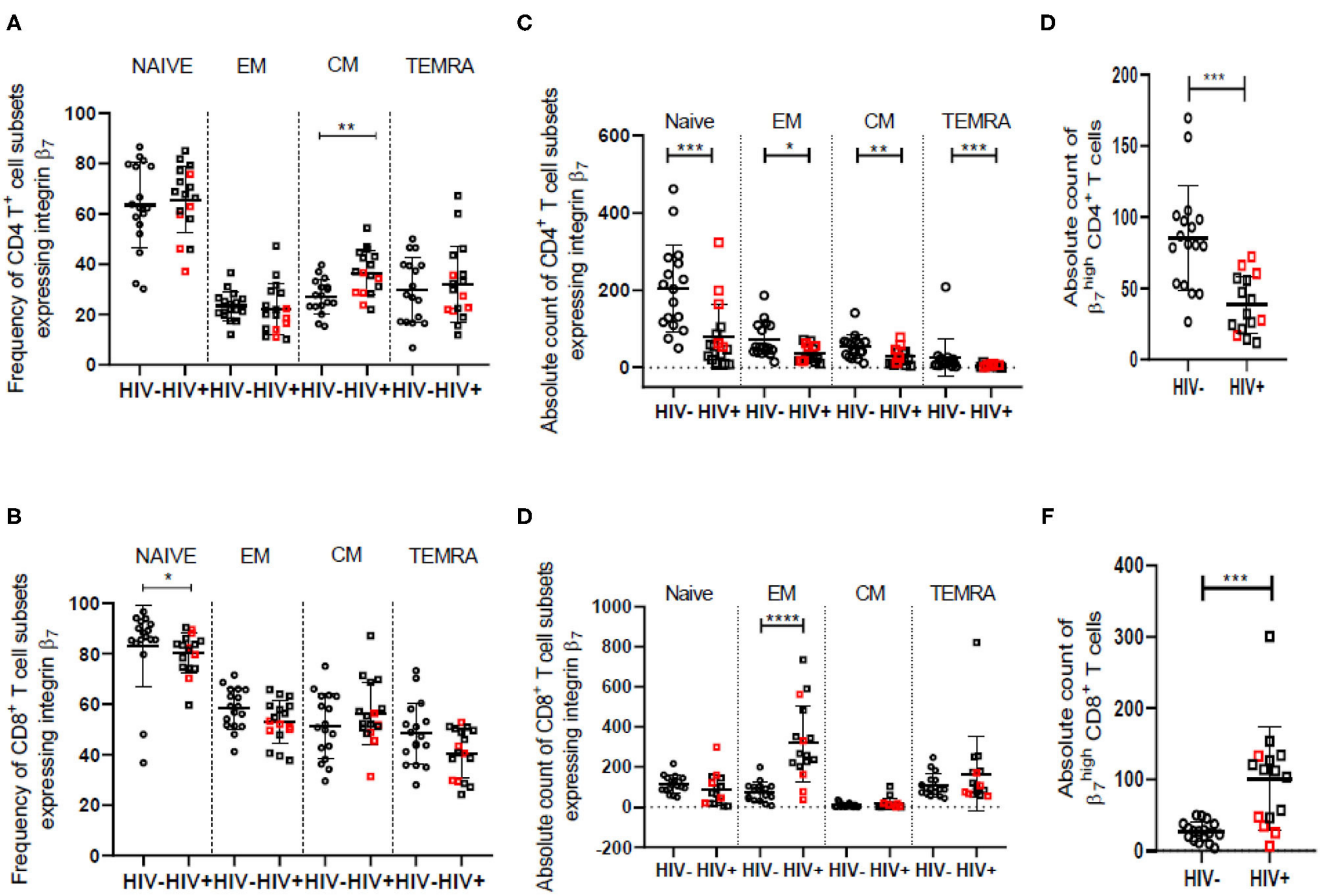
Frequency and counts of T cell subsets among HIV-uninfected (HIV^−^) and HIV infected (HIV^+^) women in peripheral blood. **(A,B)** Median frequency and distribution of cells expressing integrin α_4_β_7_ in naïve, effector memory (EM), central memory (CM) and terminally differentiated memory cells expressing RA (TEMRA) subsets of helper T cells (CD4) **(A)** and cytotoxic T cells (CD8) **(B)** among HIV^−^ (*N* = 17) and HIV^+^ (*N* = 17) women. **(C,D)** Absolute count (cells/mm^3^) of cells expressing integrin α_4_β_7_ in naïve, EM, CM, and TEMRA subsets of helper T cells (CD4) **(C)** and cytotoxic T cells (CD8) **(D)** among HIV^−^ (*N* = 17) and HIV^+^ (*N* = 16) women. **(E,F)** Median count (cells/mm^3^) and distribution of CD4^+^ T cells **(E)** and CD8^+^ T cells **(F)** having high expression of integrin β_7_ among HIV^−^ (*N* = 17) and HIV^+^ (*N* = 15) women. Median and interquartile range (error bars) are depicted. Statistical analysis was done by Mann-Whitney-test using Graphpad Prism 8. *p < 0.05, **p < 0.01, ***p < 0.001, ****p < 0.0001.

In accordance with earlier findings (42, 43), we observed a decrease in naïve and an increase in frequency of effector memory cells for both CD4^+^ and CD8^+^ T cell subsets in the HIV infected women. Absolute counts of all CD4^+^ T cell subsets were lower for HIV infected women as expected. The frequency of central memory CD8^+^ T cells was observed to be lower among HIV infected women as previously reported (43, 44). The observed lowering in the frequency of naïve and central memory CD8^+^ T cells is on account of expansion of the effector memory CD8^+^ T cell population as evident from the data on absolute counts of these cells (Supplementary Figures 6A–D). Integrin α_4_β_7_ is expressed on majority of the effector memory CD8^+^ T cells and hence the increase in absolute counts of these cells suggests that it contributes significantly toward the overall expansion of CD8^+^ T cells in the HIV infected women (Figures 3B,D).

Despite a significant drop in absolute numbers of integrin α_4_β_7_ expressing naïve and TEMRA CD4^+^ T cells, the frequency of these cells remained unchanged in HIV infected women. However, the proportion of integrin α_4_β_7_ expressing central memory CD4^+^ T cell subset is elevated in HIV infected women although there is a reduction in their absolute numbers (Figures 3A,C). Additionally, the frequency of integrin α_4_β_7_ expressing effector memory CD4^+^ T cells appears to be conserved during HIV infection. These observations indicate frequency of integrin α_4_β_7_ expressing T cells is modulated independently and often antagonistically to the frequency of the parent population. Frequency of the memory (CD45RA^−^) subset of CD4^+^ T cells having high integrin β_7_ expression ($\beta_{7}^{\text{Hi}}$ CD4^+^ T cells) is reported (13) to be greater in HIV infected individuals and correlates not just with CD4^+^ and CD8^+^ T cell activation, but also with risk of HIV and SIV acquisition and disease progression. We observed lower counts of $\beta_{7}^{\text{Hi}}$ CD4^+^ T cells among HIV infected women. Further, we report for the first time significantly higher frequency and counts of CD45RA^−^ CD8^+^ T cells expressing integrin α_4_β_7_ in HIV infected women (Figures 3E,F and Supplementary Figures 6E,F).

Our observations clearly indicate that in therapy naïve HIV infected women. there is an alteration in integrin β_7_ expressing immune cells which contribute toward HIV acquisition (endocervical T cells), anti-viral responses (CD8^+^T Cells) and formation of HIV viral reservoirs (monocytes and central memory T cells).

### Soluble MAdCAM-1 in Serum of HIV-Uninfected and HIV Infected Women

**Figure 4:**
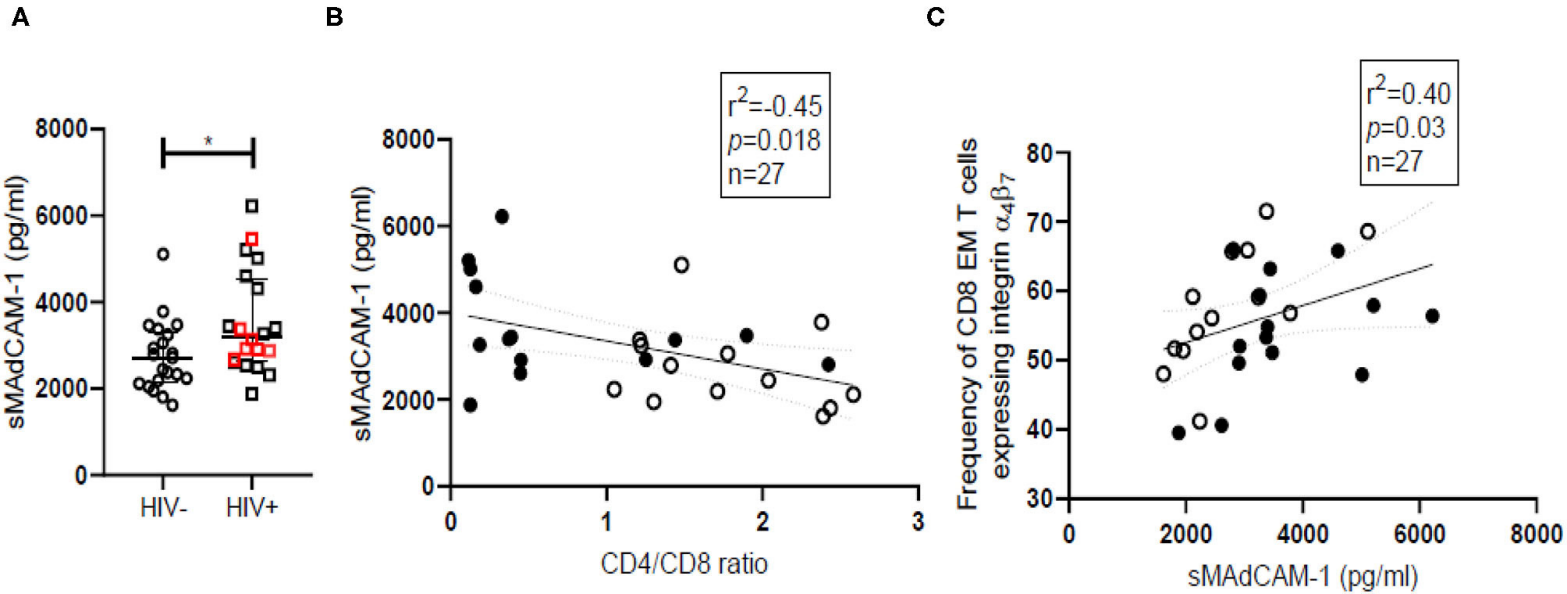
Soluble MAdCAM-1 levels and their correlation with frequency of cytotoxic effector memory cells expressing integrin α_4_β_7_ in HIV-uninfected (HIV^−^) and HIV infected (HIV^+^) women. **(A)** Distribution of sMAdCAM-1 (soluble Mucosal addressin cell adhesion molecule) levels in serum of HIV^−^ (circle; *N* = 21) and HIV^+^ (square; *N* = 20) women. Median and interquartile range (error bars) are depicted. Statistical analysis was done by Mann-Whitney test using Graphpad Prism 8 (*p < 0.05). **(B,C)** Spearman correlation analysis was performed to evaluate relation of sMAdCAM-1 with CD4/CD8 ratio **(B)** and effector memory (EM) cytotoxic T cell (CD8) **(C)** among HIV^−^ women (filled circles) and HIV^+^ women (empty circles). Linear regression with significance indicated by *p*-value and degree of association indicated by *r* square (*r*^2^) and n denotes sample size.

Soluble MAdCAM-1 (sMAdCAM-1) levels in serum have been previously associated with gut inflammation occurring during inflammatory bowel disease and have been suggested as a biomarker for tracking effectiveness of therapy (45). Since gut inflammation is a routine finding in HIV infection, we have compared sMAdCAM-1 levels in sera and observed significantly (*p* = 0.032) higher sMAdCAM-1 among HIV infected women [Median = 3,103, (IQR = 2,660–4,390)] compared to HIV-uninfected women [Median = 2,719, (IQR = 2,153–3,312)] (Figure 4A). Analysis of additional samples is required to determine if presence of HSV coinfection during HIV infection can contribute to observed differences in sMAdCAM-1 levels among HIV infected women (Supplementary Figure 7A). The negative correlation of sMAdCAM-1 levels with CD4/CD8 ratio (Figure 4B) implies an increase in sMAdCAM-1 levels upon acquisition of HIV infection. MAdCAM-1 has been reported to stimulate proliferation and activation of CD4^+^ T cells *in vitro*. However, there are no similar studies on the effect of MAdCAM-1 on CD8^+^ T cells. Although we see a negative correlation (*p* = 0.019; Spearman *r* = −0.41) between sMAdCAM-1 and the frequency of CD4^+^ T cells in the combined data of all study participants, a positive correlation (*p* = 0.01; Spearman *r* = 0.49) is observed between sMAdCAM-1 and frequency of CD8^+^ effector memory T cells expressing integrin β_7_ (CD8EMβ_7_) (Figure 4C and Supplementary Figures 8A,B). Additionally, we also observed a significant positive correlation between sMAdCAM-1 and the counts of β7^Hi^ CD8^+^ T cells (*p* = 0.005; Spearman *r* = 0.535) which are in fact memory cells (CD45RA^−^). These findings indicate that sMAdCAM-1 may play a role in driving the expansion of integrin α_4_β_7_ expressing cytotoxic T cells following HIV infection.

### TGF-β1 in Serum of HIV-Uninfected and HIV Infected Women

**Figure 5:**
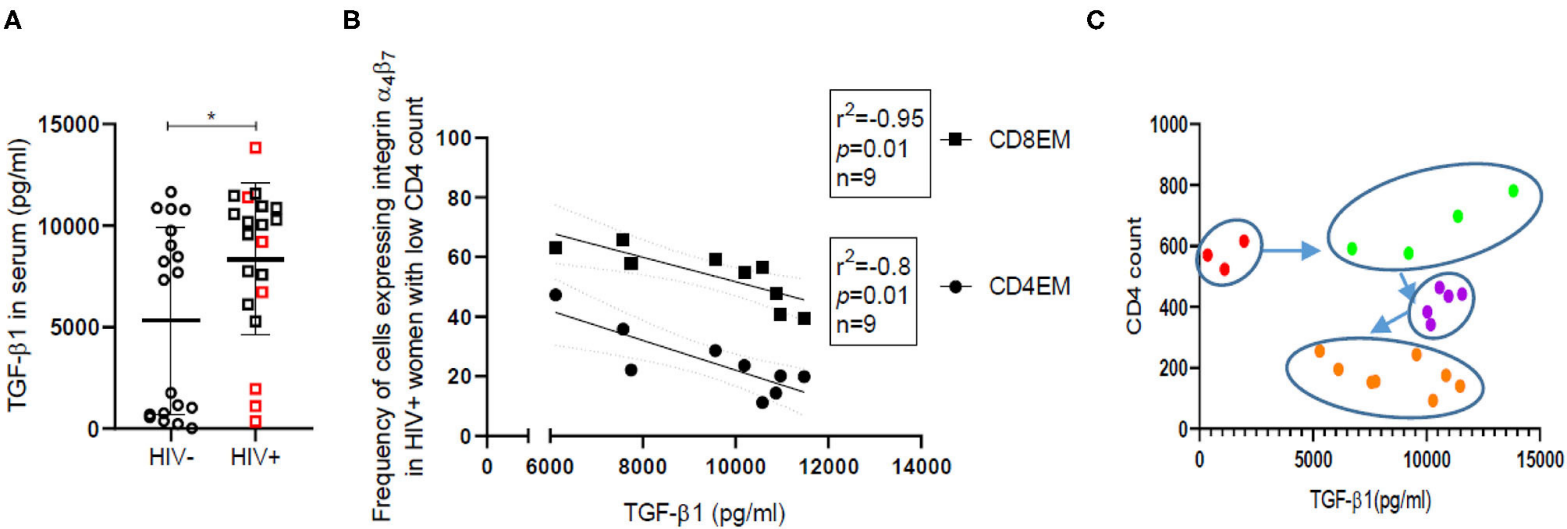
TGF-β1 levels and their correlation with frequency of effector memory cells expressing integrin α_4_β_7_ in HIV-uninfected (HIV^−^) and HIV infected (HIV^+^) women. **(A)** Distribution of TGF-β1 levels in serum of HIV-uninfected (HIV^−^) (circle) (*N* = 19) and HIV infected (HIV^+^) (square) (*N* = 20) women. Red borders indicate HIV^+^ women with CD4 count >500. Median and interquartile range (error bars) are depicted. Statistical analysis was done by Mann-Whitney *U*-test using Graphpad Prism 8 (*p < 0.05). **(B)** Spearman correlation analysis was performed to evaluate the relation of TGF-β1 levels with frequency of effector memory (EM) T cells expressing integrin α_4_β_7_ among helper (CD4EM) (circle) as well as cytotoxic T cells (CD8EM) (square). Linear regression with significance indicated by *p*-value and degree of association indicated by *r* square (*r*^2^) and n denotes sample size. **(C)** Clustering of HIV^+^ women on the basis of absolute CD4 count and TGF-β1 levels. Red dots represent women with CD4 count >500 but low TGFβ-1, while green dots represent women with CD4 count >500 and high TGF-β1. Women with CD4 count >300 are represented as purple dots and those with CD4 count <300 are represented as orange dots.

Low grade systemic inflammation among HIV infected individuals is known to be inefficiently countered by a resultant rise in immunosuppressive cytokines like TGFβ-1. We have observed a wide range of TGF-β1 levels with significantly higher (*p* = 0.04) median TGF-β1 levels (Figure 5A) in HIV infected women [Median = 9,802, (IQR = 6,272–10,942)] compared with HIV-uninfected women [Median = 7,335, (IQR = 698–9,754)]. HSV coinfection does not contribute to differences observed in TGF-β1 levels in HIV infected women (Supplementary Figure 7B). Additionally, in case of HIV infected women with low CD4 count (<500) (*n* = 9), TGF-β1 levels were found to be negatively correlated with the frequency of cells expressing CD4β7, CD8β7, CD4EMβ7, (*p* = 0.013; Spearman *r* = −0.8) and CD8EMβ7 (*p* = 0.0004; Spearman *r* = −0.95) (Figure 5B). However, these correlations don't hold true in case of HIV-uninfected participants or in HIV infected women with high CD4 count (>500). Since TGF-β1 is an anti-inflammatory cytokine, its negative correlation with different immune subsets is in line with its inhibitory function, and also indicates high TGF-β1 levels to be associated with immune dysregulation in HIV. When CD4 counts of HIV infected women were plotted against TGF-β1 levels (Figure 5C), four clusters emerged which could mimic HIV progression sequentially from high CD4 count with low TGF-β1 (red) to high CD4 count with high TGF-β1 (green). Inhibitory action of TGF-β1 perhaps results in decline of CD4^+^ T cells (purple) and ultimately at low CD4^+^ count TGF-β1 levels also recede (orange).

### Correlation Between Frequencies of Different Integrin α_4_β_7_ Expressing Immune Cell Subsets

To understand if frequencies of cells expressing integrin α_4_β_7_ are co-regulated on immune cell subsets, we performed correlation analysis which included frequencies of integrin α_4_β_7_ expressing immune cells along with demographic characteristics like age and day of sample collection post-first day of LMP as well as immunomodulatory factors in serum like sMAdCAM-1 and TGF-β1. Progesterone induced increase in expression of integrin α_4_β_7_ on CD4^+^ T cells has been reported in a previous study in macaques (46), however women using hormonal contraceptives were excluded from the study. Further, day of sample collection post-LMP was not found to be associated with frequencies of integrin α_4_β_7_ expressing immune cells in both HIV-uninfected (Supplementary Figure 8A) and HIV infected women (Supplementary Figure 8B) indicating that the changes observed may not be associated with hormonal variations that occur over the course of the menstrual cycle. Age was negatively correlated with frequencies of integrin α_4_β_7_ expressing CD4^+^ effector memory T cells and CD8^+^ effector memory T cells in HIV infected women and warrants further research to determine its significance in HIV progression.

Among HIV infected women, CD4 counts associate strongly not just with CD4/CD8 ratio but also with frequencies of naïve CD4^+^, naïve CD8^+^ and central memory CD8^+^ T cells among respective subsets signifying preservation of these cells in HIV infected individuals with high CD4 count. Its negative correlation with integrin β_7_ frequencies on CD4^+^ naïve and CD4^+^ central memory T cells imply that with decreasing CD4 counts and CD4/CD8 ratio, frequency of integrin β_7_ expressing CD4^+^ naïve and CD4^+^ central memory T cells increase in HIV infected women.

**Figure 6:**
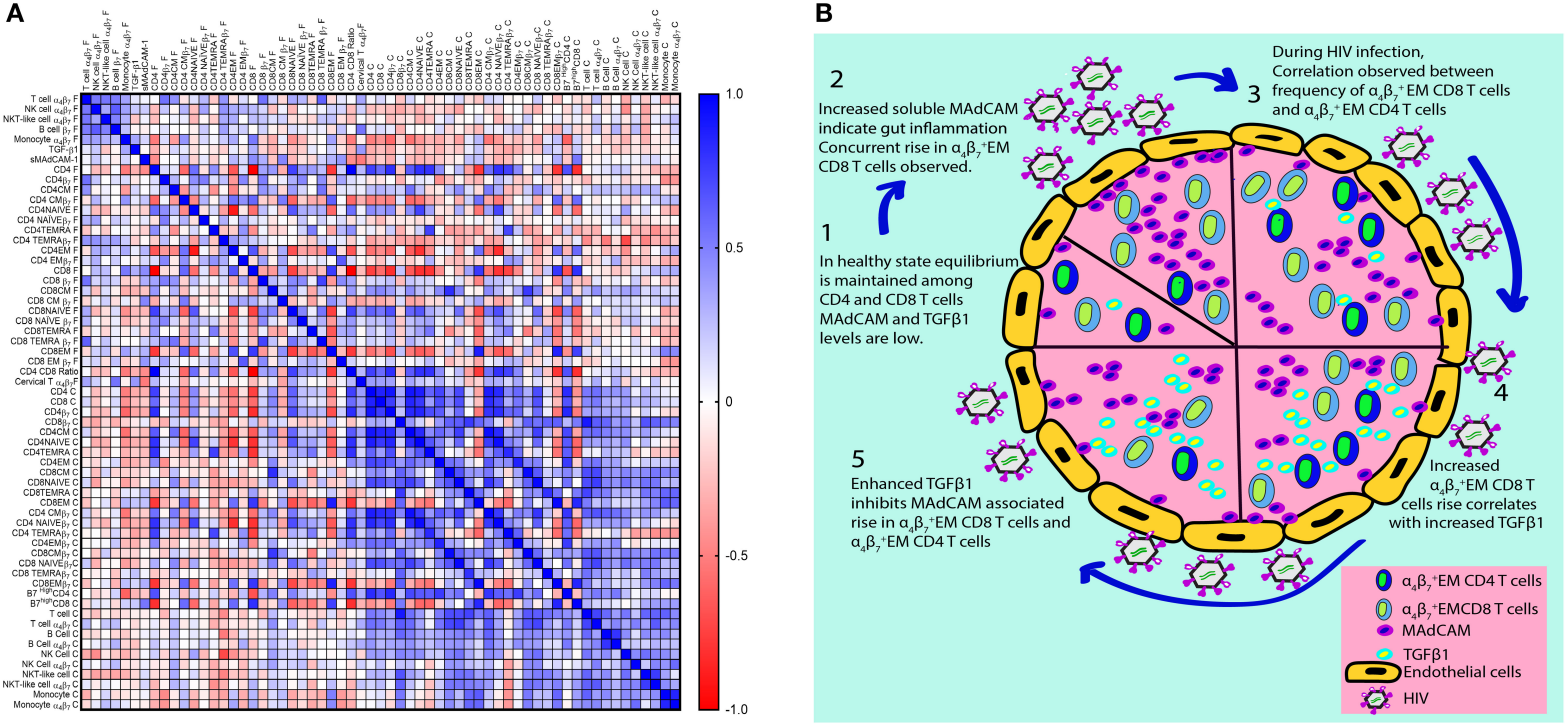
Correlation matrix for frequencies and counts of integrin α_4_β_7_ expressing immune cell subsets in HIV infected and HIV-uninfected women. **(A)** The heatmap represents pairwise Spearman correlation matrices for frequencies of integrin α_4_β_7_ cells among different immune cell subsets from both HIV infected and HIV-uninfected women. Negative correlations are indicated in red, and positive correlations are denoted in blue. **(B)** Schematic representation of HIV associated changes in integrin α_4_β_7_ expressing immune cells and their ligands as well their proposed interaction on the basis of observations from this study.

Some subsets like CD4^+^ and CD8^+^ T cells or their association with CD4/CD8 ratio have obvious correlation on account of method bias introduced during calculation of their frequencies. Yet frequencies of cells expressing integrin α_4_β_7_ remain unaffected by the frequencies of the parent population. When data of all study participants were analyzed for correlations (Figure 6A), we observed a positive correlation of varying magnitude between frequencies and counts of immune cells (Total T cells, B cells, NK cells, NKT-like cells, monocytes) expressing integrin α_4_β_7_ indicating possible co-regulated expression probably under the influence of retinoic acid.

Frequency of CD3^+^ T cells expressing integrin α_4_β_7_ correlates with CD4^+^ T cells expressing integrin α_4_β_7_ and CD8^+^ T cells expressing integrin α_4_β_7_ especially TEMRA cells expressing integrin α_4_β_7_. Integrin $\beta_{7}^{\text{Hi}}$ CD4^+^ T cells and integrin $\beta_{7}^{\text{Hi}}$ CD8^+^ T cells are strongly correlated and as previously reported (13) frequency of $\beta_{7}^{\text{Hi}}$ CD4^+^ T cell also correlates with CD4/CD8 ratio. We additionally report a strong correlation of CD4/CD8 ratio with frequency of $\beta_{7}^{\text{Hi}}$ CD8^+^ T cells that remains consistent across HIV-uninfected and HIV infected groups and could be used in future to monitor HIV disease progression or as a marker for HIV acquisition. It could even be considered to ascertain the utility of administering integrin α_4_β_7_ antibody as prophylaxis or adjunct therapy for HIV.

Frequency of monocytes (CD14) expressing integrin α_4_β_7_ increases in HIV and it correlates with other markers of HIV progression such as CD4/CD8 ratio, naïve CD8^+^ T cells and CD4^+^ central memory T cells. However, positive correlations are observed between TGF-β1 levels and frequency of CD4^+^ central memory T cells expressing integrin α_4_β_7_. Since both monocytes and CD4^+^ central memory T cells are reported to be HIV reservoirs, the potential role of TGF-β1 in giving rise to such reservoirs needs to be explored further.

sMAdCAM-1 levels are associated with frequency of CD8^+^ effector memory T cells expressing integrin α_4_β_7_ suggesting sMAdCAM-1 associated proliferation of effector memory T cells expressing integrin α_4_β_7_. Moreover, a stronger correlation exists between sMAdCAM-1 levels and the frequency as well as absolute counts of $\beta_{7}^{\text{Hi}}$ CD8^+^ effector memory T cells (Supplementary Figures 9A,B). TGF-β1 levels and frequency of CD8^+^ effector memory T cells expressing integrin α_4_β_7_ seems to have no association in the combined analysis. However, TGF-β1 level is negatively correlated with frequency of CD8^+^ effector memory T cells expressing integrin α_4_β_7_ in HIV infected women and although not statistically significant, positive association is observed in HIV-uninfected women. Literature suggests that TGF-β1 secreted by CD4^+^ T cells (47) inhibits CD8^+^ T cell proliferation. Since CD4^+^ effector memory T cells expressing integrin α_4_β_7_ and CD8^+^ effector memory T cells expressing integrin α_4_β_7_ are correlated, we put forth a novel approach to explain the T cell loss during HIV infection (Figure 6B), whereby sMAdCAM-1 mediated expansion of cytotoxic effector memory T cells drives inflammation which in turn may stimulate secretion of anti-inflammatory cytokine TGF-β1. The sMAdCAM-1 associated T cell proliferation may be responsible for maintenance of absolute CD4^+^ T cell count despite the HIV associated massive loss of T cells during early HIV infection and this may be countered by elevated levels of TGF-β1 and result in eventual decline of CD4^+^T cell count.

## Discussion

Integrin α_4_β_7_ is exclusively expressed on immune cells to facilitate gut homing by binding to MAdCAM-1 (48) which is constitutively expressed on endothelial venules of the gut. Intestinal mucosa is subjected to high antigenic exposure which is mitigated by GALT, the largest reservoir of immune cells (49). In addition to tissue resident immune cells, GALT has a constant flux of immune cells from distal sites including other mucosal surfaces via systemic blood circulation for appropriate priming of adaptive immune cells (50). This notion is affirmed by our observation that a higher frequency of integrin α_4_β_7_ expressing cells is found among adaptive immune cells compared to their innate immune counterparts in the systemic circulation. Even among T cells, naïve cells have the highest proportion of cells expressing integrin α_4_β_7_ to ensure circulation within GALT and thereby enhance the likelihood of being exposed to new antigens. Since immune surveillance is primarily carried out by circulating memory cells which perhaps have broader homing preferences and hence lower frequency of cells expressing integrin α_4_β_7_ as observed in this study. However, a subset of these cells which were CD45RA^−^ memory T cells has higher integrin β_7_ expression than naive cells. These integrin $\beta_{7}^{\text{Hi}}$ CD4^+^ T cells were reported as a marker of HIV disease progression (13) and we find integrin $\beta_{7}^{\text{Hi}}$ CD8^+^ T cells to be similarly correlated with markers of HIV progression like absolute CD4 count, CD4/CD8 ratio and changes in frequency of naïve and memory T cells.

Mucosal homing propensity of T cells expressing integrin α_4_β_7_ is evident from their higher frequency in endocervix representative of mucosal tissue resident cells, compared to blood in case of healthy controls i.e., those without HIV infection. Such tissue resident cells usually have a memory phenotype and are considered as primary targets of sexually transmitted HIV infection (17, 51). The lower frequency of integrin α_4_β_7_ expressing endocervical T cells among HIV infected women observed in this study may be on account of compartmentalized cell death of mucosal tissue resident T cells, or enhanced migration of these cells to the GALT. Additionally, downregulation of integrin α_4_β_7_ previously reported in case of SIV infection (52) could occur during HIV infection and thereby contribute toward the lower frequency of integrin α_4_β_7_ expressing endocervical T cells.

HIV infected women display a wider variation in frequencies of T cells, NK cells and NKT-like cells expressing integrin α_4_β_7_ indicating differential immune perturbation following HIV infection. Monocytes are considered to be latent reservoirs of HIV and their enhanced presence in gut perpetuates HIV transmission and replication resulting in viral persistence (53). Individuals with untreated HIV infection had enhanced frequency of innate immune cells expressing integrin α_4_β_7_. This could perhaps be on account of the host response to recruit innate immune cells to the gut in an attempt to mitigate gut HIV infection (34) which instead results in accumulation of potential viral reservoirs.

In line with previous literature, we observed an overall loss of naïve T cells (54) and enhanced frequency of effector memory cell subsets in both CD4^+^ and CD8^+^ T cells suggesting relatively higher differentiation of naïve cells to memory cells as well as inability of naïve cells to regenerate their lost repertoire during untreated HIV infection (55). This is also concordant with the increase in effector memory CD8^+^ T cells counts. However, the depletion in effector memory CD4^+^ T cell counts observed in HIV infected women despite their higher frequency suggests relative sparing compared to other CD4^+^ T cell subsets. The overall loss in CD4^+^ T cell subsets is also reflected in the integrin α_4_β_7_ expressing subpopulations. The maintenance of the frequency of integrin α_4_β_7_ expressing naïve and effector memory CD4^+^T cells indicates a differential modulation of this subpopulation compared to the parent population in the course of HIV infection. Additionally, the increased frequencies of central memory cells expressing integrin α_4_β_7_ also indicate preferential sparing of CD4^+^ T cell subpopulations that can act as HIV viral reservoirs like monocytes. Elevated levels of TGF-β1 in HIV infected individuals can also contribute toward differentiation of integrin α_4_β_7_ expressing effector memory cells to central memory cells (26) and formation of viral reservoirs. CD45RA^−^ CD4^+^ memory T cells expressing high integrin α_4_β_7_ have been previously associated with the rate of HIV acquisition and disease progression (13), increased frequency and counts of CD45RA^−^ memory $\beta_{7}^{\text{Hi}}$ CD8^+^ T cells and the depletion of the corresponding population of CD4^+^T cells observed in our study is also likely to contribute toward disease progression.

Together these innate and adaptive immune responses mounted against HIV in the gut could be responsible for gut inflammation and the subsequent damage of the GALT (56) which in turn may result in enhanced levels of sMAdCAM-1 levels in the systemic circulation of HIV infected individuals, which are reported for the first time in this study. sMAdCAM-1 levels negatively correlates with CD4/CD8 ratio that is indicative of HIV infection status. Enhanced MAdCAM levels are likely to facilitate MAdCAM-1 signaling (16) that promotes HIV infection and could drive T cell expansion and differentiation (24, 57) that may in turn lead to enhanced inflammation and a further increase in sMAdCAM-1 levels, thus propagating a continuous cycle of stimulation leading to chronic inflammation unless countered by anti-inflammatory molecules like TGF-β1. In untreated HIV infected individuals, sMAdCAM-1 as well as TGF-β1 levels are increased, and although the proportion of effector memory cells increases, the frequency of integrin α_4_β_7_ expressing cells do not alter thereby indicating the curtailing of MAdCAM-1 associated expansion of T cells.

Among HIV infected individuals with low CD4 counts, the immunosuppressive effect of TGF-β1 is evident from the negative correlation with the frequency of effector cells expressing integrin α_4_β_7_. This effect of TGF-β1 is reflected in overall CD4^+^ and CD8^+^ T cell populations as they too exhibit a negative correlation with TGF-β1 although the strength of association is weaker. Interestingly in case of HIV negative healthy individuals, TGF-β1 levels and the frequency of effector memory cells expressing integrin α_4_β_7_ are positively associated. Although not statistically significant it indicates TGF-β1 levels rise with an increase in frequency of integrin α_4_β_7_ expressing effector memory cells to perhaps control expansion of these cells. During HIV infection, in response to persistent proliferation of these cells, TGF-β1 levels rise steadily and incrementally till its immunosuppressive effect controls T cell proliferation thereby coinciding with the observed decline in CD4 T cell counts.

Based on the observations from this study, we propose that the interaction of sMAdCAM-1 with integrin α_4_β_7_ expressed on effector memory T cells may induce their expansion and thus mask HIV associated T cell death during early phase of HIV infection. However, increased TGF-β1 levels counter this proliferation of effector memory T cells and consequently HIV associated T cell death is reflected as a decline in the absolute CD4 count. The findings of our study thus highlight the need to longitudinally monitor changes in sMAdCAM-1 and TGF-β1 and simultaneously record alterations in integrin α_4_β_7_ expressing CD4^+^ and as well as CD8^+^ memory T cells in HIV infected individuals. The ambiguities reported in the targeting of integrin α_4_β_7_ for therapy in the SIV model and in case of HIV infected individuals may be explained by correlates found in our study. These correlates need to be further evaluated to determine their utility in monitoring disease progression as well as the effect of treatment with integrin α_4_β_7_ blocking antibody, which is proposed as an adjunct to ART.

## Methods

### Participant Recruitment and Sample Collection

Regular menstruating women in the age group of 18–45 years were recruited in this exploratory cross sectional study from either the integrated testing or counseling center (ICTC) at a tertiary care hospital i.e., TNMC and BYL Nair Hospital, Mumbai India, or Woman's health clinic of ICMR-NIRRH, Mumbai, India between April 2017 and December 2019. Written informed consent was taken from study participants in accordance with the study protocol approved by NIRRH Ethics Committee for Clinical Studies and ECARP, Nair Hospital. Women using hormonal contraceptives and intra-uterine contraceptive devices (IUCDs) were excluded from the study. Blood, urine, lateral vaginal wall swab and endocervical cytobrush samples were collected from antiretroviral therapy (ART) naïve HIV infected (*n* = 27) and HIV-uninfected (*n* = 21) women after a minimum of 5 days following the first day of the last menstrual period (LMP). Intravenous blood was collected in BD vacutainer K2E (EDTA) (10 ml) and SST^TM^II advance (3.5 ml). Serum was used for assessing both active and latent HSV-2 status using Calbiotech IgM and IgG ELISA kit. Two endocervical cytobrush samples were collected and transported in HiGlutaXL™ medium (Himedia) to ensure viability of immune cells for phenotyping. Vaginal swab was used for gram staining followed by Nugent scoring to determine bacterial vaginosis. HIV-uninfected women included in the study had low nugent score (<7) and were negative for HSV-2 IgM and IgG along with undetectable *Chlamydia trachomatis* DNA in the urine tested by PCR based Diagnostic kit (Ct nirrh) developed by NIRRH (ICMR).

### Immune Cell Phenotyping

Whole blood was stained with 3 panels of antibodies incubated for 20 min followed by fixation and RBC lysis using FACS lysis buffer (BD) in accordance with prescribed. Panel I consisted of integrin α4-PE (Invitrogen), integrin β7-FITC (Biolegend) CD19-PECy7 (BD Pharmingen), CD14APC (eBiosciences). Panel II consisted of integrin α4-PE (eBiosciences), integrin β7-FITC (Biolegend), CD3-PECy7 (BD Pharmingen), CD56APC (BD Pharmingen). Cells stained with both these four color panels were acquired on BD accuri C6 and analyzed using BD accuri C6 software (BD Biosciences). Panel III consisted of 5 antibodies viz. integrin β7-FITC (Biolegend), CCR7-PE (eBiosciences), CD45RA APC (Invitrogen), CD3-PECy7, CD4-PECF594 (BD Horizon). Cells stained with Panel III were acquired on FACS Aria fusion and analyzed using FlowJo VX (TreeStar, Ashland, OR,USA). Cells from the two cytobrushes were pooled and filtered through a 100-mm filter followed by staining with integrin α4-PE, integrin β7-FITC (Biolegend) and CD3-APC (BD). Viability dye 7AAD (eBiosciences) was added before acquiring the cells on BD accuri C6 flow cytometer.

### Absolute Cell Count

Liquid counting beads were added to 50 μl whole blood along with either Panel I or Panel II of antibody. Stain/lyse/no-wash protocol was followed and sample was acquired on BD Accuri^TM^ C6 flow cytometer (BD Biosciences). Data analysis was performed using BD accuri C6 software and absolute CD3 counts were used to calculate count of T cell subsets analyzed using FACSAria^TM^fusion and FlowJo VX (TreeStar, Ashland, OR, USA).

### ELISA

Two milliliter Blood was collected in SST II Advance BD Vacutainer (RWF 367956). Serum was aliquoted and stored at −80°C for batch analysis. Human MAdCAM-1 DuoSet ELISA kit (R&D Systems-DY6056-05) was used for estimation of soluble MAdCAM-1 in serum in accordance with manufacturer's protocol. Human/Mouse TGF beta 1 Uncoated ELISA kit (Invitrogen) was used for quantification of total TGF-β1 in serum. Serum was diluted 1:5 in Phosphate buffered saline and treated with 1N HCl to activate latent TGF-β1. Following 10 min incubation at room temperature, the reaction was neutralized using 1N NaOH and TGF-β1 was estimated in accordance with manufacturer's protocol.

### Statistical Analysis

Statistical analysis was performed in Graphpad prism 8. Statistical significance of differences between HIV infected and HIV-uninfected groups were assessed using Mann-Whitney-test. Spearman coefficient was used to analyze the relationship between variables and statistical significance was accepted at *p* < 0.05.

## Data Availability Statement

The original contributions generated in the study are included in the article/Supplementary Material, further inquiries can be directed to the corresponding author.

## Ethics Statement

The studies involving human participants were reviewed and approved by NIRRH Ethics Committee for Clinical Studies, ICMR-NIRRH and ECARP, NAIR Hospital. The patients/participants provided their written informed consent to participate in this study.

## Author Contributions

LS, SA, and JS provided vital support with recruitment of the study participants. VP contributed to design and execution of the flow cytometry experiments. SB acquired flow cytometry data on BD FACS Aria Fusion. NK processed the samples, analyzed the data, and interpreted the results. VB conceived the study, designed the experiments, and interpreted the data. VB and NK wrote the manuscript. VB critically reviewed the manuscript for intellectual content. All authors have read and approved the final manuscript.

## Conflict of Interest

The authors declare that the research was conducted in the absence of any commercial or financial relationships that could be construed as a potential conflict of interest.

## References

[1] Frazer IH Mackay IR Crapper RM Jones B Gust ID Sarngadharan MG. Immunotogical abnormalities in asymptomatic homosexual men: correlation with antibody to HTLV-III and sequential changes over two years. Quaterly J Med. (1986) 61:921–33. 3498182

[2] Cao W Mehraj V Kaufmann DE Li T Routy JP. Elevation and persistence of CD8 T-cells in HIV infection: the Achilles heel in the ART era. J Int AIDS Soc. (2016) 19:1–9. 10.7448/IAS.19.1.20697 26945343 PMC4779330

[3] Nokta MA Li XD Nichols J Pou A Asmuth D Pollard RB. Homeostasis of naive and memory T cell subpopulations in peripheral blood and lymphoid tissues in the context of human immunodeficiency virus infection. J Infect Dis. (2001) 183:1336–42. 10.1086/319868 11294664

[4] Shirai A Cosentino M Leitman-Klinman SF Klinman DM. Human immunodeficiency virus infection induces both polyclonal and virus-specific B cell activation. J Clin Invest. (1992) 89:561–6. 10.1172/JCI115621 1737846 PMC442888

[5] Collini P Noursadeghi M Sabroe I Miller RF Dockrell DH. Monocyte and macrophage dysfunction as a cause of HIV-1 induced dysfunction of innate immunity. Curr Mol Med. (2010) 10:727–40. 10.2174/156652410793384141 20937022

[6] Nabatanzi R Bayigga L Cose S Rowland Jones S Joloba M Canderan G. Monocyte dysfunction, activation, and inflammation after long-term antiretroviral therapy in an African cohort. J Infect Dis. (2019) 220:1414–9. 10.1093/infdis/jiz320 31323092 PMC6761975

[7] Donaghy H Gazzard B Gotch F Patterson S. Dysfunction and infection of freshly isolated blood myeloid and plasmacytoid dendritic cells in patients infected with HIV-1. Blood. (2003) 101:4505–11. 10.1182/blood-2002-10-3189 12576311

[8] Zulu MZ Naidoo KK Mncube Z Jaggernath M Goulder PJR Ndung'u T. Reduced expression of siglec-7, NKG2A, and CD57 on terminally differentiated CD56-CD16+ natural killer cell subset is associated with natural killer cell dysfunction in chronic HIV-1 clade C infection. AIDS Res Hum Retroviruses. (2017) 33:1205–13. 10.1089/aid.2017.0095 28810810

[9] Ansari AW Ahmad F Meyer-Olson D Kamarulzaman A Jacobs R Schmidt RE. Natural killer cell heterogeneity: cellular dysfunction and significance in HIV-1 immuno-pathogenesis. Cell Mol Life Sci. (2015) 72:3037–49. 10.1007/s00018-015-1911-5 25939268 PMC11113101

[10] Reynes J Portales P Segondy M Baillat V André P Réant B. CD4+ T cell surface CCR5 density as a determining factor of virus load in persons infected with human immunodeficiency virus type 1. J Infect Dis. (2000) 181:927–32. 10.1086/315315 10720514

[11] Giovannetti A Pierdominici M Mazzetta F Salemi S Marziali M Kuonen D. T cell responses to highly active antiretroviral therapy defined by chemokine receptors expression, cytokine production, T cell receptor repertoire and anti-HIV T-lymphocyte activity. Clin Exp Immunol. (2001) 124:21–31. 10.1046/j.1365-2249.2001.01502.x 11359439 PMC1906033

[12] De Roda Husman AM Koot M Cornelissen M Keet IPM Brouwer M Broersen SM. Association between CCR5 genotype and the clinical course of HIV-1 infection. Ann Intern Med. (1997) 127:882–90. 10.7326/0003-4819-127-10-199711150-00004 9382366

[13] Sivro A Schuetz A Sheward D Joag V Yegorov S Liebenberg LJ. Integrin α 4 β 7 expression on peripheral blood CD4 + T cells predicts HIV acquisition and disease progression outcomes. Sci Transl Med. (2018) 10:eaam6354. 10.1126/scitranslmed.aam6354 29367348 PMC6820005

[14] Martinelli E Tharinger H Frank I Arthos J Paital M Jr Lifson JD. HSV-2 infection of dendritic cells amplifies a highly susceptible HIV-1 cell target. PLoS Pathog. (2011) 7:e1002109. 10.1371/journal.ppat.1002109 21738472 PMC3128120

[15] Arthos J Cicala C Martinelli E Macleod K Van Ryk D Wei D. HIV-1 envelope protein binds to and signals through integrin α4β7, the gut mucosal homing receptor for peripheral T cells. Nat Immunol. (2008) 9:301–9. 10.1038/ni1566 18264102

[16] Kasarpalkar N Deb B Kumar P Bhor VM. The role of integrin α4β7 signaling in human immunodeficiency virus-1 pathogenesis and viral entry in primary CD4+ T cells as revealed by comparative phosphoproteomic signatures. OMICS J Integr Biol. (2020) 24:437–50. 10.1089/omi.2019.0196 32522079

[17] Cicala C Martinelli E McNally JP Goode DJ Gopaul R Hiatt J. The integrin α4β7 forms a complex with cell-surface CD4 and defines a T-cell subset that is highly susceptible to infection by HIV-1. Proc Natl Acad Sci USA. (2009) 106:20877–82. 10.1073/pnas.0911796106 19933330 PMC2780317

[18] Kader M Wang X Piatak M Lifson J Roederer M Veazey R. α4+β7hiCD4+ memory T cells harbor most Th-17 cells and are preferentially infected during acute SIV infection. Mucosal Immunol. (2009) 2:439–49. 10.1038/mi.2009.90 19571800 PMC2763371

[19] Guzzo C Ichikawa D Park C Phillips D Liu Q Kwon A. Virion incorporation of integrin α4β7 facilitates HIV-1 infection and intestinal homing. Sci Immunol. (2017) 2:aam7341 10.1126/sciimmunol.aam7341 28763793 PMC5653278

[20] Alzahrani J Hussain T Simar D Palchaudhuri R Abdel-Mohsen M Crowe SM. Inflammatory and immunometabolic consequences of gut dysfunction in HIV: Parallels with IBD and implications for reservoir persistence and non-AIDS comorbidities. EBioMedicine. (2019) 46:522–31. 10.1016/j.ebiom.2019.07.027 31327693 PMC6710907

[21] Mavigner M Cazabat M Dubois M L'Faqihi FE Requena M Pasquier C. Altered CD4 + T cell homing to the gut impairs mucosal immune reconstitution in treated HIV-infected individuals. J Clin Invest. (2012) 122:62–9. 10.1172/JCI59011 22156200 PMC3248296

[22] Guadalupe M Reay E Sankaran S Prindiville T Flamm J McNeil A. Severe CD4? T-cell depletion in gut lymphoid tissue during primary human immunodeficiency virus type 1 infection and substantial delay in restoration following highly active antiretroviral therapy. J Nucl Med. (2003) 77:11708–17. 10.1128/JVI.77.21.11708-11717.2003 PMC229357 14557656

[23] Mehandru S Poles MA Tenner-Racz K Manuelli V Jean-Pierre P Lopez P. Mechanisms of gastrointestinal CD4+ T-cell depletion during acuteand early human immunodeficiency virus type 1 infection. J Virol. (2007) 81:599–612. 10.1128/JVI.01739-06 17065209 PMC1797467

[24] Nawaz F Livia RG Jocelyn CR Ronke O Alia S Aftab AA. MAdCAM costimulation through Integrin-α4β7 promotes HIV replication. Mucosal Immunol. (2018) 11:1342–51. 10.1038/s41385-018-0044-1 29875402 PMC6160318

[25] Goes LR Sajani A Sivro A Olowojesiku R Ray JC Perrone I. The V2 loop of HIV gp120 delivers costimulatory signals to CD4 + T cells through Integrin α 4 β 7 and promotes cellular activation and infection. Proc Natl Acad Sci USA. (2020) 117:32566–73. 10.1073/pnas.2011501117 33288704 PMC7768698

[26] Cheung K-W Wu T Ho SF Wong YC Liu L Wang H. α4β7+CD4+ effector/effector memory T cells differentiate into productively and latently infected central memory T cells by transforming growth factor β1 during HIV-1 Infection. J Virol. (2018) 92:e01510–17. 10.1128/JVI.01510-17 29386290 PMC5874435

[27] Byrareddy SN Arthos J Cicala C Villinger F Ortiz KT Little D. Sustained virologic control in SIV+ macaques after antiretroviral and α4β7 antibody therapy. Science. (2016) 354:197–202. 10.1126/science.aag1276 27738167 PMC5405455

[28] Byrareddy SN Kallam B Arthos J Cicala C Nawaz F Hiatt J. Targeting α4β7 integrin reduces mucosal transmission of simian immunodeficiency virus and protects gut-associated lymphoid tissue from infection. Nat Med. (2014) 20:1397–400. 10.1038/nm.3715 25419708 PMC4257865

[29] Ansari AA Reimann KA Mayne AE Takahashi Y Stephenson ST Wang R. Blocking of α4β7 gut-homing integrin during acute infection leads to decreased plasma and gastrointestinal tissue viral loads in simian immunodeficiency virus-infected rhesus macaques. J Immunol. (2011) 186:1044–59. 10.4049/jimmunol.1003052 21149598 PMC3691699

[30] Iwamoto N Mason RD Song K Gorman J Welles HC Arthos J. Blocking a4b7 integrin binding to SIV does not improve virologic control. Science. (2019) 365:1033–6. 10.1126/science.aaw7765 31488690 PMC9513815

[31] Wittner M Schlicker V Libera J Bockmann JH Horvatits T Seiz O. Comparison of the integrin α4β7 expression pattern of memory T cell subsets in HIV infection and ulcerative colitis. PLoS ONE. (2019) 14:1–23. 10.1371/journal.pone.0220008 31356607 PMC6663001

[32] Schweighoffer T Tanaka Y Tidswell M Erle DJ Horgan KJ Luce GEG. Selective expression of integrin α4β7 on a subset of human CD4+ memory T cells with hallmarks of gut-trophism. J Immunol. (1993) 151:717–29. 7687621

[33] Shannon B Yi TJ Thomas-Pavanel J Chieza L Janakiram P Saunders M. Impact of asymptomatic herpes simplex virus type 2 infection on mucosal homing and immune cell subsets in the blood and female genital tract. J Immunol. (2014) 192:5074–82. 10.4049/jimmunol.1302916 24760150

[34] Allers K Fehr M Conrad K Epple HJ Schürmann D Geelhaar-Karsch A. Macrophages accumulate in the gut mucosa of untreated HIV-infected patients. J Infect Dis. (2014) 209:739–48. 10.1093/infdis/jit547 24133185

[35] Jelicic K Cimbro R Nawaz F Huang DW Zheng X Lempicki RA. HIV-1 gp120 impairs B cell proliferation by inducing TGF-β1 production and FcRL4 expression. Nat Immunol. (2014) 14:1–25. 10.1038/ni.2746 24162774 PMC3870659

[36] Reitano KN Kottilil S Gille CM Zhang X Yan M O'Shea MA. Defective plasmacytoid dendritic cell-NK cell cross-talk in HIV infection. AIDS Res Hum Retroviruses. (2009) 25:1029–37. 10.1089/aid.2008.0311 19795986 PMC2828160

[37] Reeves RK Evans TI Gillis J Wong FE Kang G Li Q. SIV Infection Induces Accumulation of plasmacytoid dendritic cells in the gut mucosa. J Infect Dis. (2012) 206:1462–8. 10.1093/infdis/jis408 22711907 PMC3529602

[38] Pereira LE Onlamoon N Wang X Wang R Li J Reimann KA. Preliminary *in vivo* efficacy studies of a recombinant rhesus anti- α4 β7 monoclonal antibody. Cell Immunol. (2009) 259:165–76. 10.1016/j.cellimm.2009.06.012 19616201 PMC2765715

[39] Erle DJ Briskin MJ Butcher EC Lazarovits AI Tidswell M. Expression and function of the MAdCAM-1 receptor, integrin alpha 4 beta 7, on human leukocytes. J Immunol. (1994) 153:517–28. 7517418

[40] Wang X Xu H Gill AF Pahar B Kempf D Rasmussen T. Monitoring α4β7 integrin expression on circulating CD4+ T cells as a surrogate marker for tracking intestinal CD4+ T cell loss in SIV infection. Mucosal Immunol. (2009) 2:518–26. 10.1038/mi.2009.104 19710637 PMC3702381

[41] Gumbi PP Jaumdally SZ Salkinder AL Burgers WA Mkhize NN Hanekom W. CD4 T cell depletion at the cervix during HIV infection is associated with accumulation of terminally differentiated T cells. J Virol. (2011) 85:13333–41. 10.1128/JVI.05671-11 21994461 PMC3233181

[42] Messele T Abdulkadir M Fontanet AL Petros B Hamann D Koot M. Reduced naive and increased activated CD4 and CD8 cells in healthy adult Ethiopians compared with their Dutch counterparts. Clin Exp Immunol. (1999) 115:443–50. 10.1046/j.1365-2249.1999.00815.x 10193416 PMC1905237

[43] Singh AK Salwe S Padwal V Velhal S Sutar J. Delineation of homeostatic immune signatures defining viremic non-progression in HIV-1 infection. Front Immunol. (2020) 11:182. 10.3389/fimmu.2020.00182 32194543 PMC7066316

[44] Breton G Chomont N Takata H Ahlers J Filali-mouhim A Riou C. Programmed death-1 is a marker for abnormal distribution of naive/memory T cell subsets in HIV-1 infection. J Immunol. (2013) 191:2194–204. 10.4049/jimmunol.1200646 23918986 PMC3815464

[45] Paul S Williet N Di Bernado T Berger AE Boschetti G Filippi J. Soluble mucosal addressin cell adhesion molecule 1 and retinoic acid are potential tools for therapeutic drug monitoring in patients with inflammatory bowel disease treated with vedolizumab: a proof of concept study. J Crohn's Colitis. (2018) 12:1089–96. 10.1093/ecco-jcc/jjy077 29860366

[46] Goode D Truong R Villegas G Calenda G Guerra-perez N Piatak M. HSV-2-driven increase in the expression of α4β7 correlates with increased susceptibility to vaginal SHIV SF162P3 infection. PLoS Pathog. (2014) 10:e1004567. 10.1371/journal.ppat.1004567 25521298 PMC4270786

[47] Donkor MK Sarkar A Li MO. TGF-β1 produced by activated CD4+ T cells antagonizes T cell surveillance of tumor development. Oncoimmunology. (2012) 1:162–71. 10.4161/onci.1.2.18481 22720237 PMC3376999

[48] Gorfu G Rivera-Nieves J Ley K. Role of beta7 integrins in intestinal lymphocyte homing and retention. Curr Mol Med. (2009) 9:836–50. 10.2174/156652409789105525 19860663 PMC2770881

[49] Delgobo M Paludo KS Fernandes D de Oliveira JG Ortolan GL Favero GM. Gut: key element on immune system regulation. Brazilian Arch Biol Technol. (2019) 62:1–14. 10.1590/1678-4324-2019180654

[50] Shaw SK Brenner MB. The β7 integrins in mucosal homing and retention. Semin Immunol. (1995) 7:335–42. 10.1016/1044-5323(95)90014-4 8580465

[51] Joag VR Mckinnon LR Liu J Kidane ST Yudin MH Nyanga B. Identification of preferential CD4+T-cell targets for HIV infection in the cervix. Mucosal Immunol. (2016) 9:1–12. 10.1038/mi.2015.28 25872482

[52] Budde ML Lhost JJ Dudley DM Rakasz EG Connor DHO. Integrin α4β7 is downregulated on the surfaces of simian immunodeficiency virus SIVmac239-infected cells. J Virol. (2010) 84:6344–51. 10.1128/JVI.00430-10 20410278 PMC2903238

[53] Campbell Jennifer H Hearps AC Martin GE Williams KC Crowe SM. The importance of monocytes and macrophages in HIV pathogenesis, treatment, and cure. AIDS. (2014) 28:2175–87. 10.1097/QAD.0000000000000408 25144219 PMC6331181

[54] Di Mascio M Sereti I Matthews LT Natarajan V Adelsberger J Lempicki R. Naïve T-cell dynamics in human immunodeficiency virus type 1 infection: effects of highly active antiretroviral therapy provide insights into the mechanisms of Naïve T-cell depletion. J Virol. (2006) 80:2665–74. 10.1128/JVI.80.6.2665-2674.2006 16501076 PMC1395465

[55] Benveniste O Flahault A Rollot F Elbim C Estaquier J Pédron B. Mechanisms involved in the low-level regeneration of CD4+ cells in HIV-1-infected patients receiving highly active antiretroviral therapy who have prolonged undetectable plasma viral loads. J Infect Dis. (2005) 191:1670–9. 10.1086/429670 15838794

[56] Arroyo-mercado F Likhtshteyn M Huynh CD Chokshi T. CD4 levels and viral load during ulcerative colitis flares in patients with human immunodeficiency virus: a case series. Am J Med Case Rep. (2019) 7:162–6. 10.14309/01.ajg.0000597964.48914.5131511837

[57] Andrews OE Van Ryk D Ansari A Cicala C Fauci A Arthos J. MAdCAM as a potential biomarker of HIV-induced inflammation. J Immunol. (2016) 196.

