# Supplementary material for: Frequency of Effector Memory Cells Expressing Integrin α4β7 Is Associated With TGF-β1 Levels in Therapy Naïve HIV Infected Women With Low CD4+ T Cell Count
Source: Front Immunol. 2021 Mar 22;12:651122. doi: 10.3389/fimmu.2021.651122 (PMC8019712; doi:10.3389/fimmu.2021.651122)
Supplement: Supplementary file 1 [file Data_Sheet_1.PDF]

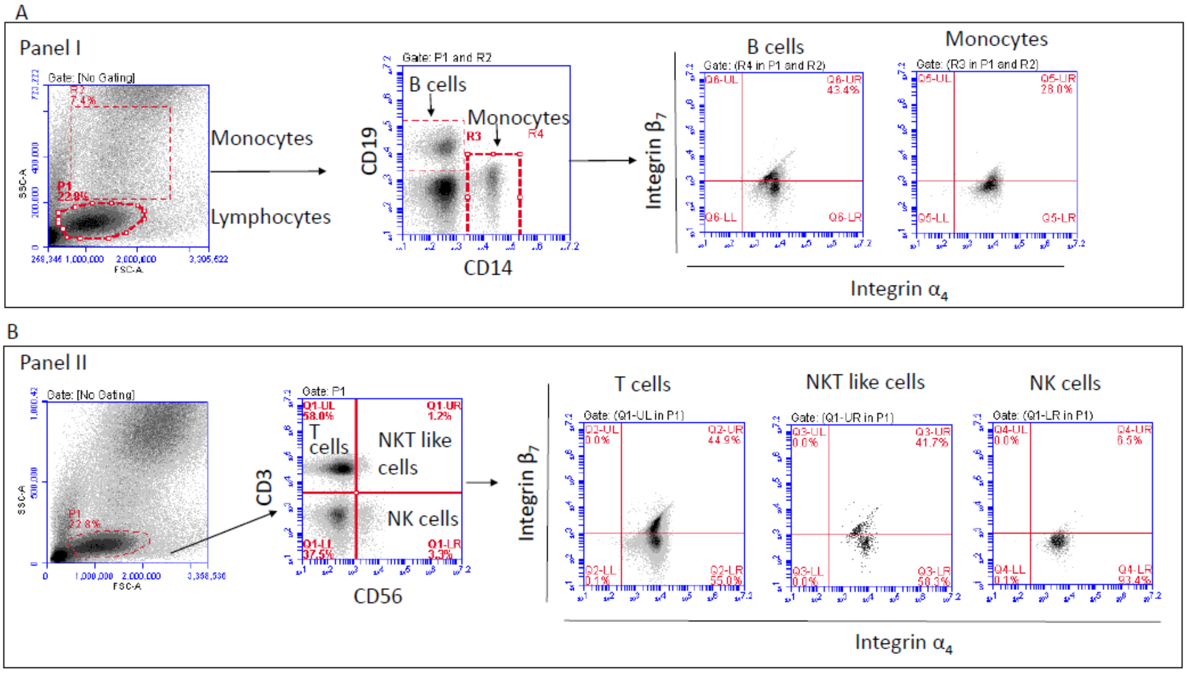

S1 Gating strategy and representative plots of peripheral blood immune cells analysed using BD accuri C6 flow cytometer. Integrin α_4 and β_7 was identified using two independent antibodies and cells expressing both were termed as positive of integrin $\alpha_4\beta_7$. Since the flow cytometer analysis was limited to 4 fluorophores, two panels were designed to evaluate major immune cell subsets. (A) Overall gating strategy for analysis of B cells and monocytes. Cells were gated on lymphocytes and monocytes based on Forward scatter, FSC and side scatter, SSC. CD19 and CD14 was respectively used as makers for B cells and monocytes (B) Overall gating strategy for T cells, NK cells and NKT cells identified using markers $CD3^+CD56^-$ for T cells, $CD3^+CD56^+$ for NKT and $CD3^-CD56^+$ for NK cells.

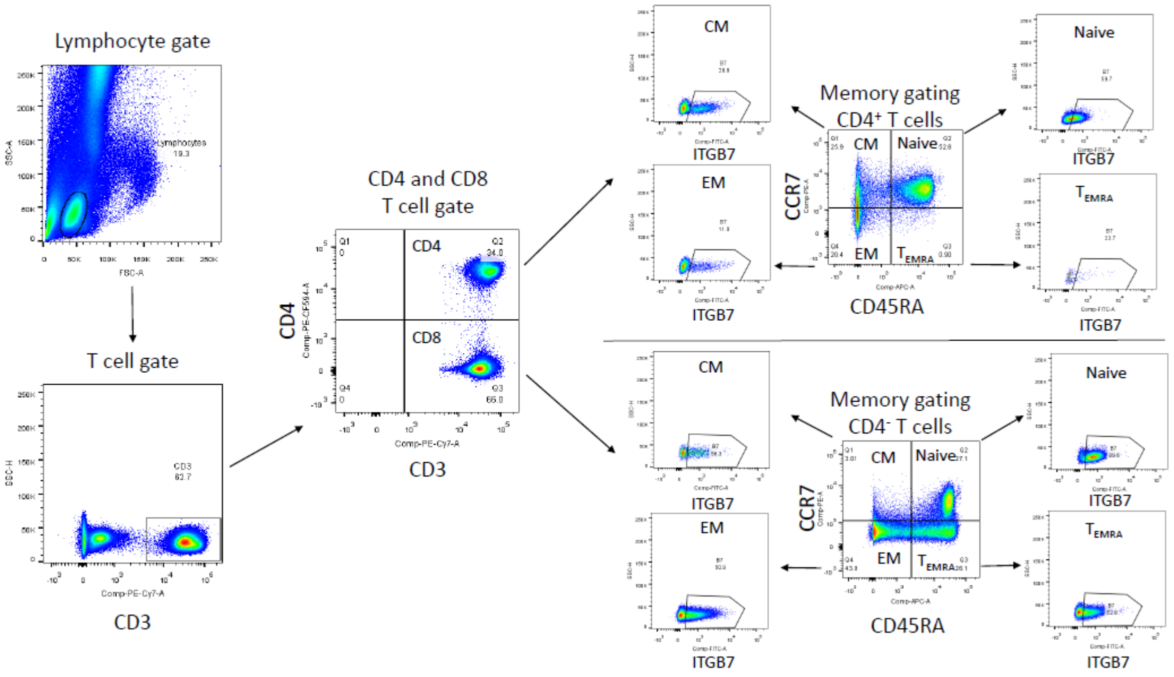

S2 Gating strategy for the analysis of peripheral T cells subsets expressing integrin β$_7$. Whole blood was stained with antibodies, fixed and acquired on BD FACS Aria. Representative gating strategy demonstrated using HIV sample. Lymphocytes were gated on the basis of forward scatter, FSC and side scatter, SSC. CD3+CD4+ cells were termed as CD4 T cells and CD3+CD4- cells are represented as CD8 T cells. CCR7 and CD45RA was used to define Naïve cells, Effector memory cells (EM), Central memory cells (CM) and terminally differentiated memory cells expressing RA(TEMRA) cells.

Gating strategy for $\beta 7^{high}$ memory cells

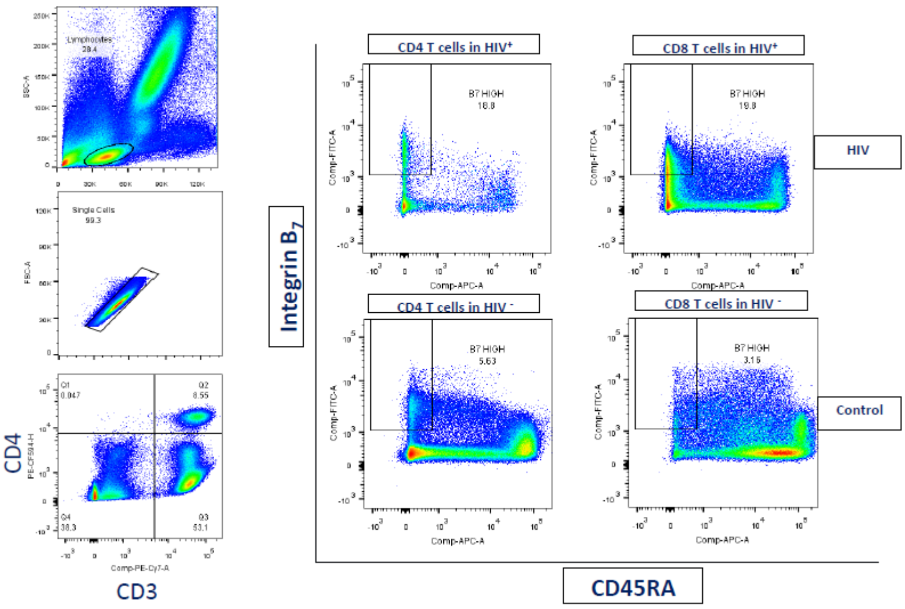

S3 Gating strategy for the analysis of T cells having high expression of integrin β_7. Gating strategy similar to Sivro, et.al. 2018, is represented for HIV$^+$ sample. Samples acquired using BD FACS Aria were analysed to study cells having high expression of integrin β_7. Lymphocytes were gated on the basis of forward scatter, FSC and side scatter, SSC. FSC-A and FSC-H parameters were used for singlet selection. CD3+CD4+ cells were termed as CD4 T cells and CD3+CD4- cells are represented as CD8 T cells. Representative comparison of HIV$^+$ and HIV$^-$ frequency of β_7^{high} T cells.

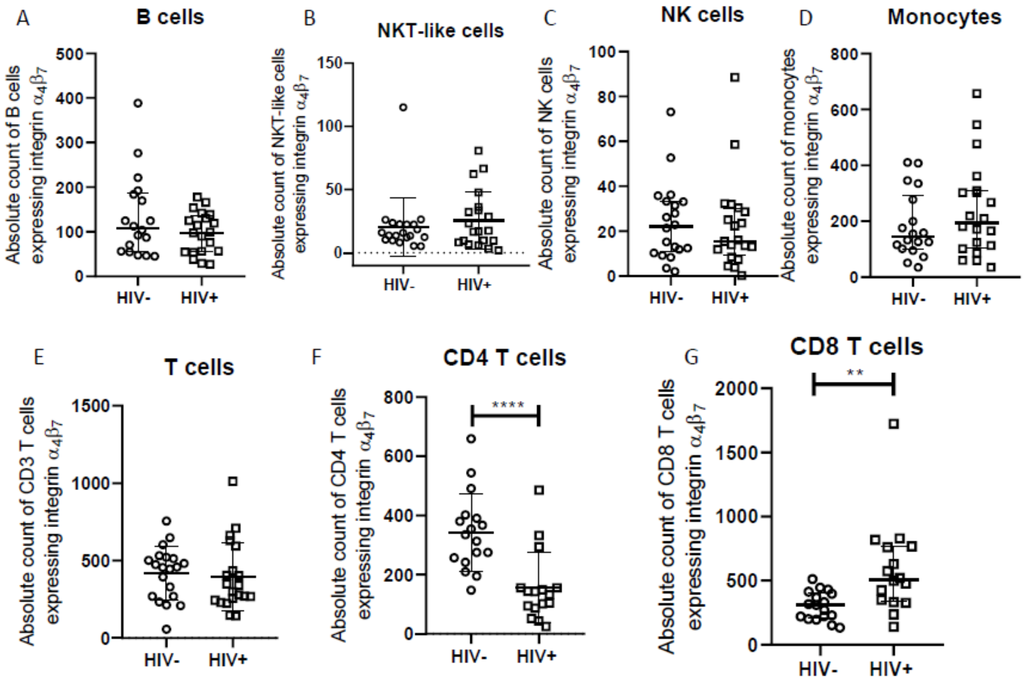

S4. Comparison of absolute count(cells/mm^3) of immune cell expressing integrin $\alpha_4\beta_7$ among HIV-uninfected (HIV-) and HIV infected (HIV+) women. A-E. Absolute count (cells/mm^3) of integrin $\alpha_4\beta_7$ expressing B cells (A), Natural killer (NK)cells (B), NK T-like (NKT) (C) cells, monocytes (D) and CD3+CD56- T cells (E) in peripheral blood of HIV-(N=18) and HIV+(N=20) women is compared. Absolute count (cells/mm^3) of CD3+CD4+ T cell(F) and CD3+CD4-(CD8)(G) T cells expressing integrin $\alpha_4\beta_7$ among HIV-(N=17) women is represented with a circle and square represents HIV+ women HIV-(N=16).

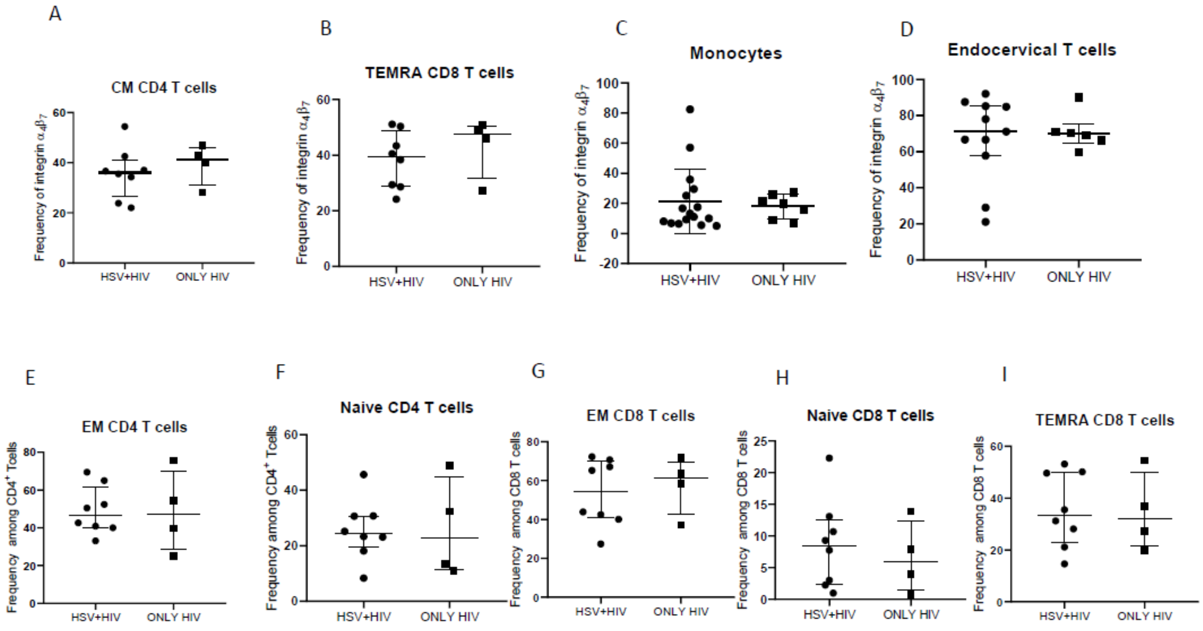

S5. Frequency of immune cells among HIV seropositive women with latent HSV-2 coinfection. A-D. Frequency of cells expressing integrin $\alpha_4\beta_7$ in Central memory (CM) (A), T_{EMRA} (B), monocytes(C) and endocervical T cells(D) are compared in HIV seropositive women with latent HSV-2 coinfection(HSV+HIV) and women with HIV infection(only HIV) without IgG antibodies against HSV-2 in sera as tested by ELISA. **E-I.** Frequency of Effector memory (EM), Naïve and T_{EMRA} cells in subsets of Helper $CD4^+$ T cell and Cytotoxic $CD8^+$ T cell. Statistical analysis done by Wilcoxon matched-pairs signed rank test using Graphpad Prism 8.

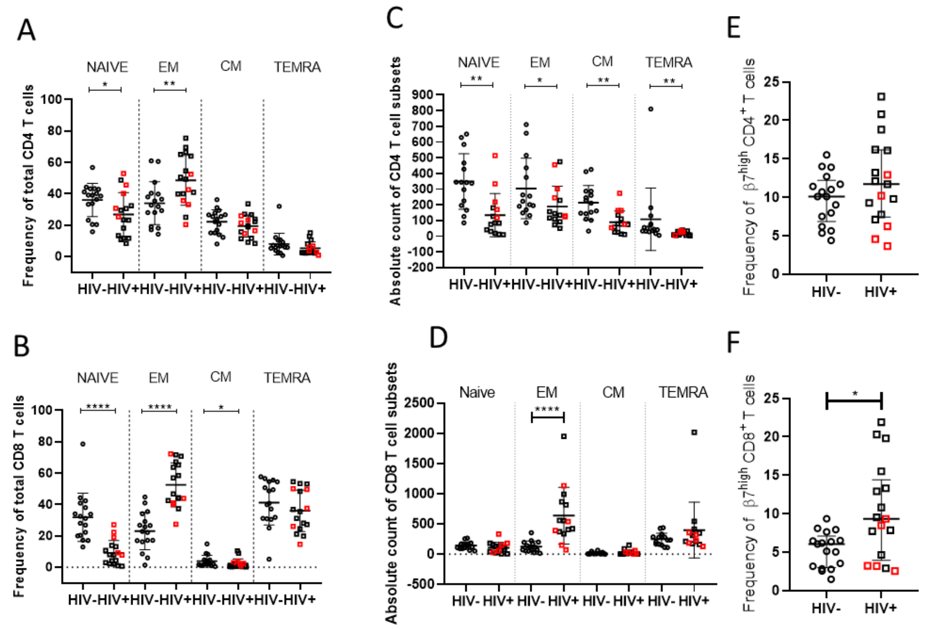

S6. Frequency of T cell subsets among HIV-uninfected (HIV-) and HIV infected (HIV+) women. A-B. Distribution of Naïve cells, Effector memory cells(EM),Central memory cells(CM) and terminally differentiated memory cells expressing RA(TEMRA) in peripheral blood among HIV-(N=17) and HIV+(N=17) women in Helper T cells(CD4) (A) and Cytotoxic T cells(CD8)(B). **C-D.** Absolute count (cells/mm^3) of Helper T (B) cell)and Cytotoxic T (D)cell subsets among HIV-(N=17) and HIV+(N=16). **E-F.** Median frequency and distribution of CD4 T cells (E) and CD8 T cells (F) having high expression of integrin β_7 in HIV-(N=17) and HIV+(N=15). Statistical analysis done by Mann-Whitney using Graphpad Prism 8. * p<0.05, ** p<0.001, **** p<0.0001.

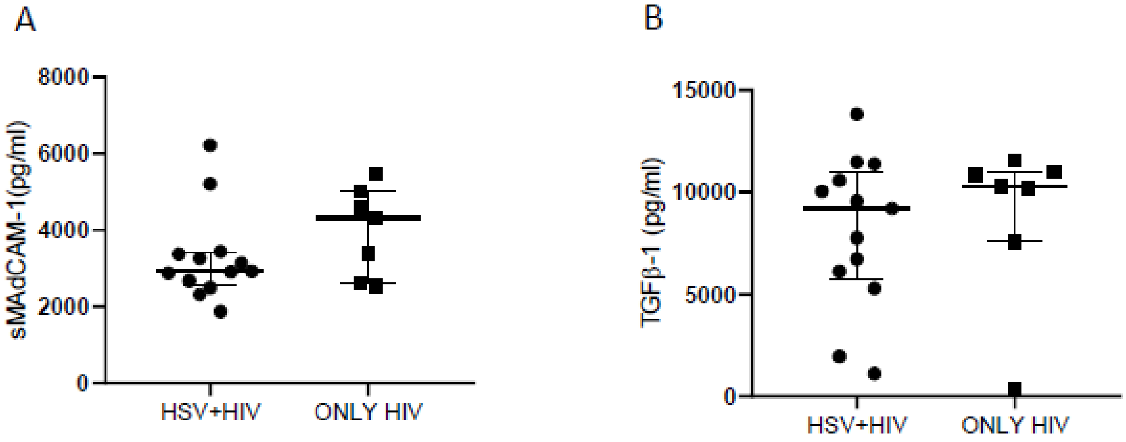

S7. Distribution of sMAdCAM-1 and TGF-β1 in HIV seropositive women with latent HSV-2 coinfection. Distribution of sMAdCAM-1 (A)and TGF-β1(B) among HIV seropositive women with latent HSV-2 coinfection (HSV+HIV) and women with HIV infection(only HIV) without IgG antibodies against HSV-2 in sera as tested by ELISA. Error bars indicate median and interquartile range. Statistical analysis done by Mann-Whitney test using Graphpad Prism 8

A

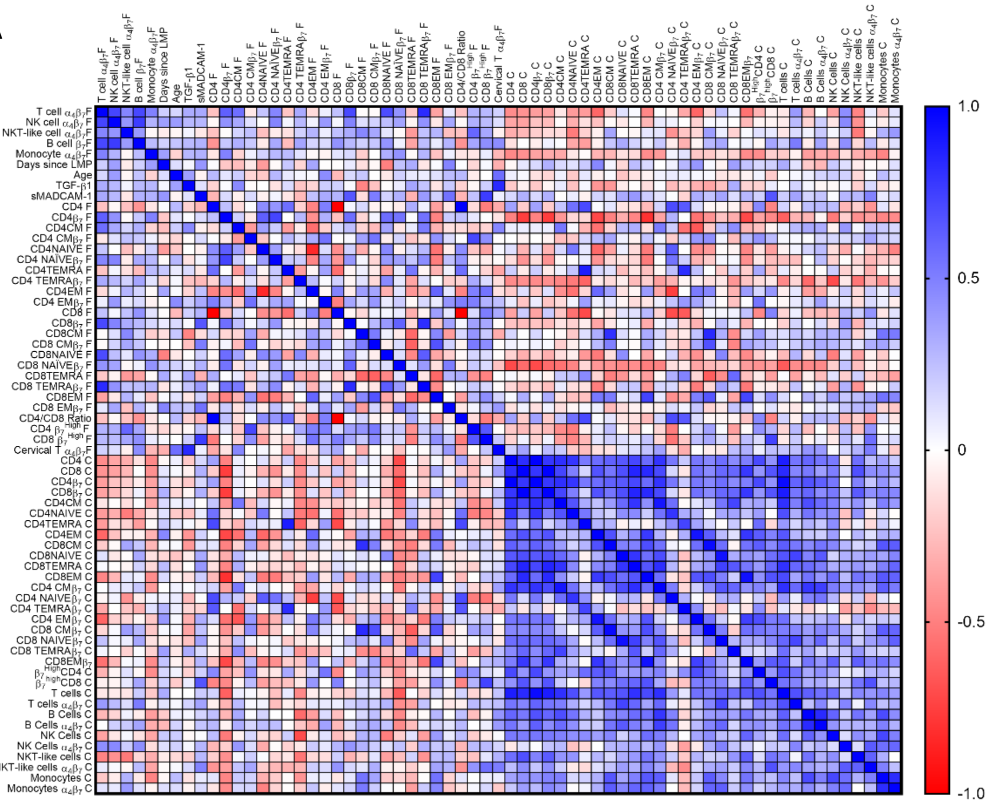

B

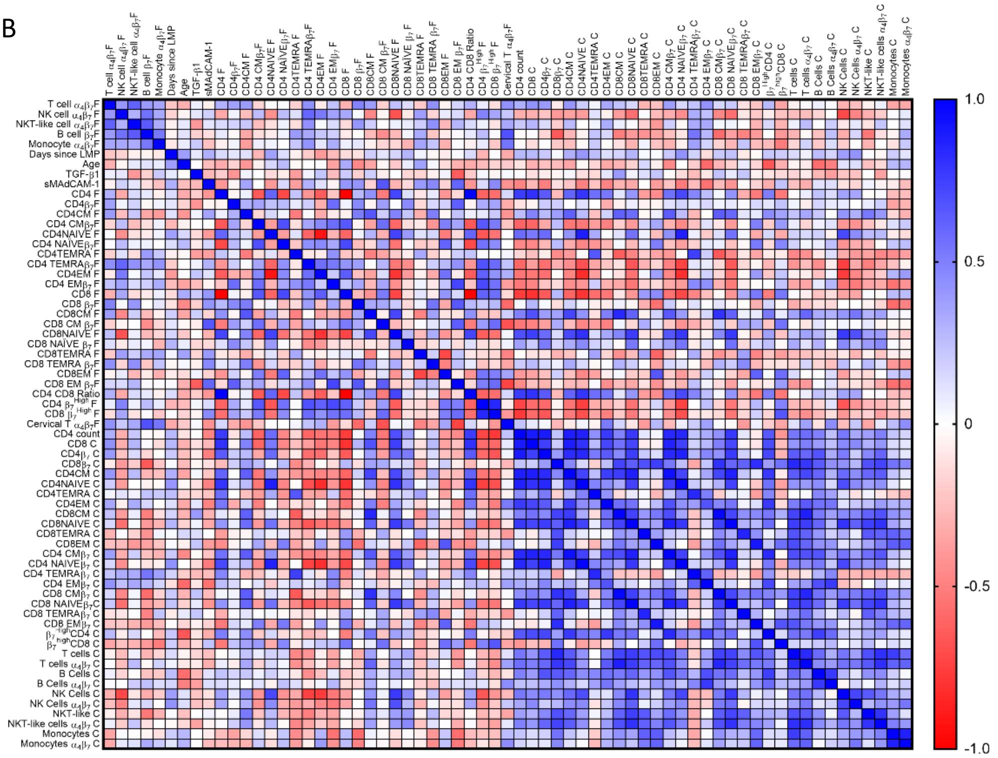

S8. Correlation matrix for β7 frequencies among immune cells from study participants. The correlation heatmap represents pairwise Spearman correlation matrices of β_7 frequencies among immune cells for HIV seronegative (A) and HIV seropositive(B) women along with a combined heatmap of all study participants

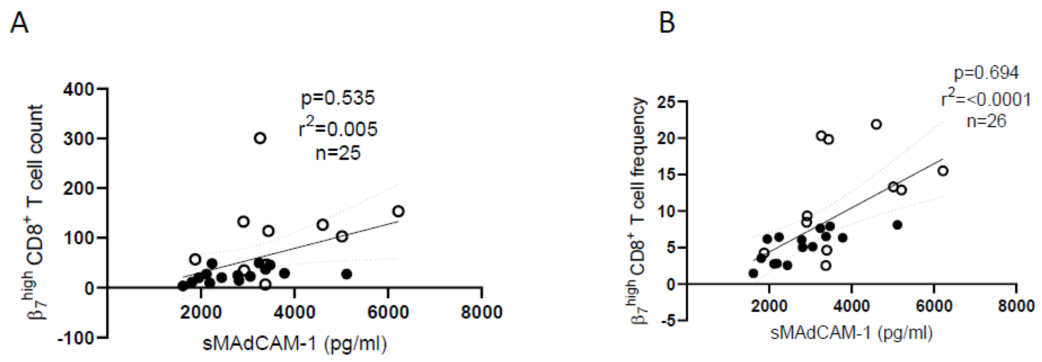

S9. Association of sMAdCAM-1 with β_7^{high} CD8$^+$ T cells. Spearman correlation analysis performed to analyze association of sMAdCAM-1 with (A)count (cell/mm^3) and (B)frequency of β_7^{high} CD8$^+$ T cells. HIV- women depicted with black circles and HIV+ women depicted using hollow circles. Linear regression with significance represented by p value and degree of association represented by r square (r^2) is depicted while n represents sample size.
